# Alterations in urinary ceramides, sphingoid bases, and their phosphates among patients with kidney disease

**DOI:** 10.3389/fneph.2024.1343181

**Published:** 2024-03-07

**Authors:** Yoshifumi Morita, Eri Sakai, Hideaki Isago, Yoshikazu Ono, Yutaka Yatomi, Makoto Kurano

**Affiliations:** ^1^ Department of Clinical Laboratory, The University of Tokyo Hospital, Tokyo, Japan; ^2^ Department of Clinical Laboratory Medicine, Graduate School of Medicine, The University of Tokyo, Tokyo, Japan

**Keywords:** ceramide, sphingosine-1-phosphate, sphingolipids, urine, machine learning

## Abstract

**Background:**

To avoid an invasive renal biopsy, noninvasive laboratory testing for the differential diagnosis of kidney diseases is a desirable goal. As sphingolipids are demonstrated to be involved in the pathogenesis of various kidney diseases, we investigated the possible usefulness of the simultaneous measurement of urinary sphingolipids for differentiating kidney diseases.

**Materials and methods:**

Residual urine specimens were collected from patients who had been clinically diagnosed with chronic glomerulonephritis (CGN), diabetic mellitus (DM), systemic lupus erythematosus (SLE), and arterial hypertension (AH). The urinary sphingolipids—CERs C16:0, C18:0, C18:1, C20:0, C22:0, and C24:0; sphingosine [Sph]; dihydrosphingosine; sphingosine 1-phosphate [S1P]; and dihydroS1P [dhS1P]—were measured by liquid chromatography–tandem mass spectrometry. Based on the results, machine learning models were constructed to differentiate the various kidney diseases.

**Results:**

The urinary S1P was higher in patients with DM than in other participants (*P* < 0.05), whereas dhS1P was lower in the CGN and AH groups compared with control participants (*P* < 0.05). Sph and dhSph were higher in patients with CGN, AH, and SLE than in those with control participants (*P* < 0.05). The urinary CERs were significantly higher in patients with CGN, AH, and SLE than in those with control participants (*P* < 0.05). As a results of constructing a machine learning model discriminating kidney diseases, the resulting diagnostic accuracy and precision were improved from 94.03% and 66.96% to 96.10% and 78.26% respectively, when the urinary CERs, Sph, dhSph, S1P, dhS1P, and their ratios were added to the models.

**Conclusion:**

The urinary CERs, sphingoid bases, and their phosphates show alterations among kidney diseases, suggesting their potential involvement in the development of kidney injury.

## Background

Urinalysis, including urine chemistry and urinary sediments, are the diagnostic methods typically used for the diagnosis of kidney diseases. However, the specificities of those laboratory findings are low, and a renal biopsy is sometimes required. Given the invasive nature of a biopsy, developing novel noninvasive laboratory tests for the differential diagnosis of kidney diseases remains an important task.

A series of elegant basic studies recently suggested that sphingolipids are promising urinary biomarkers for kidney diseases (1, 2). The sphingolipids are composed of a hydrophobic and a hydrophilic chain, with the various fatty acid components of each sphingolipid determining its species (3). The ceramides (CERs) and sphingosine 1-phosphate (S1P) are well-known sphingolipid metabolites. Ceramide can be converted to sphingosine by ceramidase (4, 5), and sphingosine can be converted to S1P by sphingosine kinases (6). S1P can either be converted back to sphingosine (Sph) or irreversibly degraded by S1P lyase (7). Through five kinds of S1P receptors, S1P exerts potent biologic effects (8–10). Dihydrosphingosine 1-phosphate (dhS1P) is an analog for the S1P receptors that is produced from dihydrosphingosine (dhSph), and dihydroceramides can process dhSph into CERs (11). **Supplementary Figure S1** presents a schematic of the foregoing metabolic pathways (5, 12–14).

The biologic functions of sphingolipids have been widely studied. CER is involved in the stress response, apoptosis, inflammation, and proliferation (15–18), and it contributes to the pathogenesis of various diseases, including diabetes mellitus (DM) (19), neurodegenerative disorders (20), multiple sclerosis (21), and coronary artery disease (22). S1P plays an important role in the regulation of cellular processes such as anti-apoptosis, proliferation, and vasoprotection (23), and it has been reported to have protective properties in atherosclerosis and cardiomyocytes (24–26).

Sphingolipids have been proposed to play an important role in the pathogenesis of acute kidney injury (27–29) or chronic kidney disease (30, 31) through their modulating effects on inflammation and the immune system. In addition, CER has been shown to be involved in the apoptosis of glomerular mesangial and tubular cells (32). In contrast, S1P receptors are expressed in glomerular mesangial cells, glomerular endothelial cells, tubular cells, and podocytes; and S1P has been demonstrated to play a protective role in renal injury and fibrosis (33–37).

To date, publications regarding the alterations in levels of the CERs and S1P in the urine are limited. In individuals with chronic kidney disease, our group and others have demonstrated that urinary CERs is associated with diabetic nephropathy (38, 39). Recently, our group also reported that urinary sphingolipids are dynamically changed in individuals with COVID-19, depending on the severity of the infection and any ensuing renal complications (40).

Considering that background, we focused on the presence of sphingolipids in urinary specimens and attempted to develop a noninvasive liquid chromatography–tandem mass spectrometry assessment useful for the differential diagnosis of kidney diseases.

## Materials and methods

### Participants

Patients who had been clinically diagnosed with chronic glomerulonephritis (CGN, n = 126), DM (n = 167), systemic lupus erythematosus (SLE, n = 88), and arterial hypertension (AH) (n = 39) were enrolled into the study. Patients who were renal transplant recipients or who were undergoing hemodialysis were excluded. The group of CGN included the patients with IgA nephropathy, IgA vasculitis, anti-neutrophil cytoplasmic antibody (ANCA)- associated nephritis, and other glomerulonephritis. We have designated patients with DM as a distinct group separate from CGN because it is clinically important to distinguish the cause of renal dysfunction by diabetic nephropathy and other CGN. Since lupus nephritis, which is the primary complication of SLE, necessitates specialized treatment by rheumatologists and it is also important to distinguish lupus nephritis from other forms of glomerulonephritis, the patients with SLE were also analyzed as a separate group. Patients with AH were identified in cases where they had hypertension, and other specific diseases were excluded. After clinical laboratory testing, residual urine samples from those patients were collected and centrifuged at 1,700*g* for five minutes. The resulting supernatant was stored at −80°C. A group of 80 individuals without kidney disease, with a urinary total protein (U-TP) level below 0.15 g/gCr, and with an estimated glomerular filtration rate (eGFR) greater than 60 mL/min per 1.73 m^2^ were enrolled as control participants. **Table 1**; **Supplementary Table S1** presents the characteristics of the study participants.

**Table 1:** Clinical characteristics of participants.

	control	CGN	DM	AH	SLE	*P* value
n	80	126	167	39	88	
Biopsy, n	0 (0.0%)	65 (51.6%)	5 (3.0%)	2 (5.1%)	35 (39.8%)	
Age (years)	58.7±11.7	54.2±18.7	67.5±13.5^*, †^	71.5±11.1^*, †^	50.3±15.5^*, ‡, §^	<0.001
Sex (M/F)	45/35	64/62	113/54	36/3	12/76	<0.001
GFR grade, n						<0.001
1	6 (7.5%)	10 (8.3%)	14 (8.4%)	0 (0.0%)	15 (17.2%)	
2	74 (92.5%)	27 (22.3%)	58 (34.7%)	4 (10.5%)	35 (40.2%)	
3	0 (0.0%)	56 (46.3%)	55 (32.9%)	17 (44.7%)	34 (39.1%)	
4	0 (0.0%)	21 (17.4%)	23 (13.8%)	15 (39.5%)	2 (2.3%)	
5	0 (0.0%)	7 (5.8%)	17 (10.2%)	2 (5.3%)	1 (1.1%)	
eGFR	74.7±10.5	51.1±25.7^*^	51.3±26.5^*^	35.3±16.5^*, †, ‡^	66.2±24.7^*, †, ‡, §^	<0.001
U-TP (g/gCr)	0.02 [0.00, 0.04]	0.55 [0.34, 1.18] ^*,^	0.23 [0.08, 1.84] ^*, †^	0.44 [0.15, 1.24] ^*,^	0.22 [0.08, 0.72] ^*, †^	<0.001
S1P (nmol/mgCr)	0.11 [0.00, 0.39]	0.24 [0.13, 0.47] ^*^	0.62 [0.44, 1.44] ^*, †^	0.25 [0.14, 0.39] ^‡^	0.25 [0.09, 0.47] ^‡^	<0.001
dhS1P (nmol/mgCr)	5.02 [0.00, 18.24]	0.17 [0.00, 4.08] ^*^	3.30 [0.16, 7.54]	0.00 [0.00, 6.27] ^*, ‡^	1.26 [0.62, 2.51]	<0.001
Sphingosine (ng/mgCr)	0.47 [0.27, 0.95]	1.26 [0.61, 2.47] ^*^	0.50 [0.17, 1.78] ^†^	0.96 [0.60, 2.22] ^*^	2.07 [1.24, 3.96] ^*, †, ‡, §^	<0.001
dhSph (ng/mgCr)	0.14 [0.08, 0.23]	0.39 [0.16, 1.16] ^*^	0.43 [0.25, 0.98] ^*^	0.39 [0.18, 1.56] ^*^	0.46 [0.25, 0.92] ^*^	<0.001
CER C16:0 (ng/mgCr)	1.20 [0.53, 1.89]	1.62 [1.05, 3.57] ^*^	0.79 [0.31, 3.01] ^†^	2.09 [1.21, 5.03] ^*, ‡^	2.67 [1.58, 4.63] ^*, ‡^	<0.001
CER C18:0 (ng/mgCr)	0.20 [0.10, 0.30]	0.34 [0.18, 0.92] ^*^	0.16 [0.06, 0.71] ^†^	0.44 [0.26, 1.31] ^*, ‡^	0.55 [0.30, 0.82] ^*, ‡^	<0.001
CER C18:1 (ng/mgCr)	0.03 [0.02, 0.07]	0.08 [0.03, 0.34] ^*^	0.04 [0.02, 0.14]	0.08 [0.05, 0.55] ^*, ‡^	0.14 [0.06, 0.27] ^*, ‡^	<0.001
CER C20:0 (ng/mgCr)	0.52 [0.27, 1.14]	0.46 [0.22, 1.17]	0.24 [0.09, 1.21]	1.00 [0.49, 1.89] ^‡^	0.50 [0.37, 1.01] ^‡^	<0.001
CER C22:0 (ng/mgCr)	0.98 [0.41, 1.87]	0.75 [0.40, 2.47]	0.29 [0.12, 1.42] ^*, †^	1.29 [0.59, 2.68] ^‡^	1.20 [0.84, 2.36] ^‡^	<0.001
CER C24:0 (ng/mgCr)	1.42 [0.59, 2.25]	1.69 [0.98, 4.63]	0.67 [0.28, 2.28] ^†^	1.58 [1.10, 4.65] ^‡^	3.44 [2.09, 5.20] ^*, †, ‡^	<0.001
CER C16:0/C24:0	0.82 [0.68, 0.94]	0.97 [0.69, 1.27] ^*^	1.07 [0.93, 1.24] ^*^	1.06 [0.75, 1.36] ^*^	0.80 [0.67, 1.06] ^‡, §^	<0.001
CER C18:0/C16:0	0.18 [0.14, 0.24]	0.20 [0.15, 0.27]	0.19 [0.16, 0.22]	0.21 [0.15, 0.26]	0.19 [0.14, 0.24]	0.596
CER C18:0/C24:0	0.16 [0.12, 0.22]	0.20 [0.12, 0.28]	0.22 [0.18, 0.25] ^*^	0.21 [0.16, 0.28] ^*^	0.15 [0.12, 0.19] ^†, ‡, §^	<0.001
CER C18:1/C18:0	0.23 [0.13, 0.38]	0.26 [0.14, 0.39]	0.32 [0.20, 0.42]	0.25 [0.16, 0.42]	0.24 [0.16, 0.42]	0.093
CER C22:0/C20:0	1.55 [1.25, 2.15]	1.83 [1.46, 2.22]	1.16 [1.03, 1.55] ^*, †^	1.62 [1.07, 2.35]	2.21 [1.78, 2.55] ^*, †, ‡, §^	<0.001
CER C24:0/C20:0	2.38 [1.76, 3.66]	3.82 [2.32, 6.24] ^*^	3.04 [2.39, 3.70] ^†^	2.54 [1.51, 4.69]	6.24 [3.88, 7.68] ^*, †, ‡, §^	<0.001

Data was presented as mean±SD for age and eGFR, and median [interquartile range] for the other variables. Abbreviations: AH, arterial hypertension; CGN, chronic glomerulonephritis; CER, ceramide; dhS1P, dihydrosphingosine 1-phosphate; dhSph, dihydrosphingosine; DM, diabetes mellitus; SLE, systemic lupus erythematosus; S1P, sphingosine 1-phosphate; U-TP, urinary total protein.

*: P < 0.05 vs. control, †: P < 0.05 vs. chronic glomerulonephritis, ‡: P < 0.05 vs. diabetes mellitus, §: P < 0.05 vs. arterial hypertension.

### Liquid chromatography–tandem mass spectrometry

Urinary levels of the sphingolipids of interest were measured by liquid chromatography–tandem mass spectrometry methods on an LC-8060 high-performance liquid chromatograph triple quadrupole mass spectrometer (Shimadzu, Kyoto, Japan) as previously described and validated (38, 41).

### Urinalysis

The urine sediment analysis was performed by manual microscopy in compliance with the guideline published by Japanese Committee for Clinical Laboratory Standards. Red and white blood cells and epithelial cells (squamous epithelial cells, urothelial cells, and renal tubular epithelial cells) were counted per high-power field of view. Urinary casts were classified into hyaline casts, granular casts, epithelial casts, fatty casts, red blood cell casts, white blood cell casts, vacuolar denatured casts, crystal casts, fibulin casts, broad casts, and waxy casts. The numbers of casts and oval fat bodies (OFBs) were counted per whole field. Urinary creatinine was measured using an enzyme assay (L-Type Wako Creatinine M: Fujifilm Wako Pure Chemical Corporation, Osaka, Japan), and U-TP was measured using the pyrogallol red method (Micro TP-Test Wako: Fujifilm Wako Pure Chemical Corporation).

### Statistical analysis

The data analysis was performed using the IBM SPSS Statistics software application and SPSS Modeler (version 18.0: IBM, Armonk, NY, USA), SIMCA (MKS Umetrics, Umeå, Sweden), and MetaboAnalyst (version 5.0: https://www.metaboanalyst.ca/). The median was used in the analysis and a Kruskal–Wallis test, followed by a Steel–Dwass *post-hoc* test, was used to examine differences in urinary sphingolipid levels between groups of study participants. In SIMCA, a principal component analysis was conducted to classify the kidney diseases in patients based on their urinary sphingolipid results. Using SPSS Modeler, machine learning models for the differential diagnosis of the kidney diseases were constructed. Graphics presenting the results were prepared using GraphPad Prism 9 (GraphPad Software, San Diego, CA, USA), SIMCA, or MetaboAnalyst. Statistical significance was accepted at a *P* value less than 0.05 in all analyses.

## Results

### Alterations in ceramides, sphingoid bases, and their phosphates by kidney disease

Differences in the levels of the urinary sphingolipids among patients with kidney diseases and control participants were investigated (Tabel 1). Urinary S1P was observed to be higher in patients with DM than in other participants and higher in patients with CGN than in control participants (**Supplementary Figure 2A**). Urinary dhS1P was observed to be lower in patients with CGN or AH than in control participants (**Supplementary Figure 2B**). Urinary Sph was observed to be higher in patients with SLE than in other patients, and higher in patients with CGN than in patients with DM or in control participants (**Supplementary Figure 2C**). Urinary dhSph was observed to be higher in patients with CGN, DM, AH, or SLE than in control participants (**Supplementary Figure 2D**). Of the CER species, urinary C16:0, C18:0, C20:0, C22:0, and C24:0 were observed to be significantly higher in patients with CGN, AH, or SLE than in patients with DM, and CER C18:1 was observed to be higher in patients with AH or SLE than in patients with DM or in control participants (**Supplementary Figures 2E-J**).

### Correlations of urinary ceramides and sphingosine 1-phosphate with urinary casts, oval fat bodies, urinary protein, and eGFR

The results of correlation analysis are shown in **Supplementary Figure S2K**. Urinary S1P was positively correlated with OFBs (*r*_s_ = 0.38, *P* < 0.01), hyaline casts (*r*_s_ = 0.31, *P* < 0.01), granular casts (*r*_s_ = 0.31, *P* < 0.01), epithelial casts (*r*_s_ = 0.31, *P* < 0.01), vacuolar denatured casts (*r*_s_ = 0.14, *P* < 0.01), fatty casts (*r*_s_ = 0.34, *P* < 0.01), WBC casts (*r*_s_ = 0.12, *P* < 0.05), waxy casts (*r*_s_ = 0.27, *P* < 0.01), U-TP (*r*_s_ = 0.54, *P* < 0.01), and negatively correlated with eGFR (*r*_s_ = −0.25, *P* < 0.01). Similarly, urinary sphingosine and dhSph showed positive correlations with OFBs (*r*_s_ = 0.34, *P* < 0.01 and *r*_s_ = 0.27, *P* < 0.01), hyaline casts (*r*_s_ = 0.23, *P* < 0.01 and *r*_s_ = 0.25, *P* < 0.01), granular casts (*r*_s_ = 0.33, *P* < 0.01 and *r*_s_ = 0.27, *P* < 0.01), epithelial casts (*r*_s_ = 0.23, *P* < 0.01 and *r*_s_ = 0.18, *P* < 0.01), vacuolar denatured casts (*r*_s_ = 0.10, *P* < 0.05 and *r*_s_ = 0.11, *P* < 0.05), fatty casts (*r*_s_ = 0.26, *P* < 0.01 and *r*_s_ = 0.20, *P* < 0.01), waxy casts (*r*_s_ = 0.24, *P* < 0.01 and *r*_s_ = 0.19, *P* < 0.01), U-TP (*r*_s_ = 0.47, *P* < 0.01 and *r*_s_ = 0.46, *P* < 0.01), and negatively correlated with eGFR (*r*_s_ = −0.21, *P* < 0.01 and *r*_s_ = −0.22, *P* < 0.01). Urinary CERs were positively correlated with OFBs (*r*_s_ = 0.25 to 0.35), U-TP (*r*_s_ = 0.31 to 0.44), and urinary casts excepted with vacuolar denatured casts and RBC casts, whereas CER C18:1 showed no significant correlation with epithelial casts and WBC casts in addition to above. Meanwhile, urinary dhS1P was negatively correlated with hyaline casts (*r*_s_ = −0.16, *P* < 0.01).

When performing a correlation analysis by the disease group, only S1P in the AH group, sphingosine, dhSph and CERs in the DM group, and CER C16:0 and C18:0 in the CGN group showed negative correlation with eGFR. Besides, the urinary sphingosine, dhSph, and CERs were not correlated with U-TP in the AH group (**Supplementary Table S2**).

### Receiver operating characteristic analyses to differentiate kidney diseases

**Figure 1:**
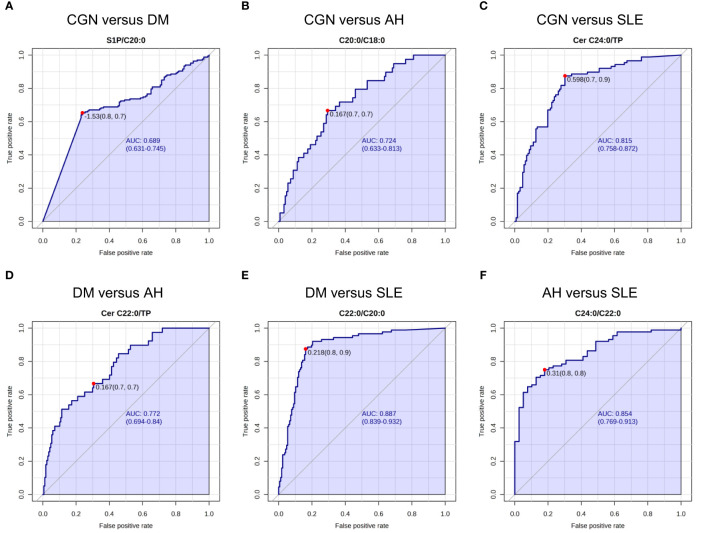
Receiver operating characteristic (ROC) analysis for the urinary sphingolipids. The representative areas under the receiver operating characteristic curves (AUROCCs) differentiate the various kidney diseases. **(A)** The ratio of sphingosine 1-phosphate (S1P) to the ceramide (CER) C20:0 differentiates patients with chronic glomerulonephritis (CGN) from those with diabetes mellitus (DM). **(B)** The ratio of the CER C20:0 to the CER C18:0 differentiates patients with CGN from those with AH. **(C)** The ratio of the CER C24:0 to urinary total protein (U-TP) differentiates patients with CGN from those with systemic lupus erythematosus (SLE). **(D)** The ratio of the CER C22:0 to U-TP differentiates patients with DM from those with AH. **(E)** The ratio of the CER C22:0 to the CER C20:0 differentiates patients with DM from those with SLE. **(F)** The ratio of the CER C24:0 to the CER C22:0 differentiates patients with AH from those with SLE.

We next used a curve analysis to validate the usefulness of urinary sphingolipids for differentiating kidney diseases. **Supplementary Table S3** presents the resulting areas under the receiver operating characteristic curve (AUROCCs). The ratios of S1P to CERs and of CER C22:0 to CER C20:0 were selected as the significant variables for differentiating patients with CGN from patients with DM (AUROCCs: 0.663–0.684), followed by hematuria and dipstick glucose. For differentiating patients with CGN from those with AH, the C20:0 to C18:0 ratio, the C20:0 value, and the ratios of C20:0 and C22:0 to U-TP were selected (AUROCCs: 0.689–0.721). The ratios of CER species to U-TP were selected to differentiate patients with CGN from those with SLE (0.750–0.816), those with DM from those with AH (0.732–0.770), and those with DM from those with SLE (0.812–0.886). **Figure 1** presents representative ROC curves. The results suggest that measurement of a single sphingolipid cannot distinguish kidney diseases better than traditional urinalysis, except for SLE versus CGN, SLE versus DM, and DM versus AH.

### Principal component analysis

**Figure 2:**
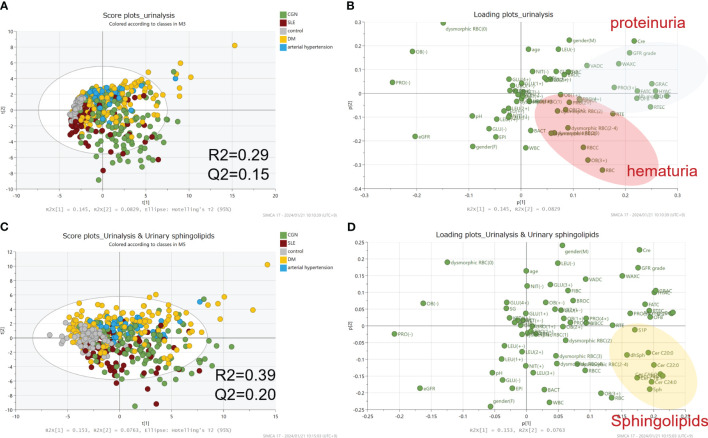
Principal component analysis for the differential diagnosis of kidney diseases. The principal component analysis results are visualized as score plots **(A, C)** and loading plots **(B, D)**. The upper-panel plots represent data from clinical laboratory tests alone; the lower-panel plots represent the addition of the data from measurements of the urinary sphingolipids.

We next used principal component analysis to investigate whether the comprehensive measurement of urinary CERs, sphingoid bases, and their phosphates contributes to the differentiation of kidney diseases. When only clinical data, including urine sediment results, were used in the model, R2 and Q2 were 0.29 and 0.15. The R2 and Q2 improved to 0.39 and 0.20 when urinary sphingolipids were added (**Figure 2**). Those results suggest that urinary CERs, sphingoid bases, and their phosphates measurements could potentially contribute to the differentiation of kidney diseases, without being sufficient to differentiate specific kidney diseases.

### A machine learning model for the differential diagnosis of kidney diseases

**Figure 3:**
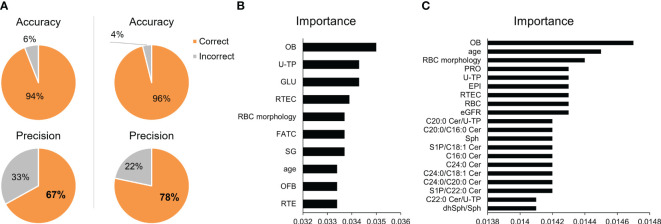
Validation of the machine learning models. **(A)** Pie charts depict the accuracy and precision achieved in differentiating kidney diseases with the use of machine learning models. The left panels present the results of models that used only standard clinical laboratory data. The right panels present the results of models that added the urinary sphingolipid measurements. The top 10–20 important variables for prediction are shown for **(B)** the models using only standard clinical laboratory data and **(C)** the models that added the urinary sphingolipid measurements.

Finally, we constructed a machine learning model for the differential diagnosis of kidney diseases and investigated its usefulness. The algorithms for the machine learning model included artificial neural networks, decision trees, support vector machines, regression analysis, and Bayesian networks. Three optical models were selected. When only clinical laboratory data were used, with selection of the XGBoost Tree, Random Tree, and Classification and Regression Tree models, an accuracy (positive predictive rate in training data) of 94.03% and a precision (positive predictive rate in testing data) of 66.96% were achieved. When urinary sphingolipids measured in the present study and their ratios were added, with selection of the XGBoost Tree, Neural Networks, and Linear Support Vector Machines, the accuracy and precision improved to 96.10% and 78.26% respectively (**Supplementary Table S4**; **Figure 3A**). **Figures 3B, C** presents the top 10–20 important variables. Although clinical laboratory data contributed more to the diagnosis of renal diseases, urinary sphingolipids clearly made the diagnoses more accurate.

## Discussion and conclusions

In the present study, we observed that urinary CERs, sphingoid bases, and their phosphates are significantly changed depending on the kidney disease that is present. To develop a noninvasive alternative to the renal biopsy for diagnosing kidney diseases, we constructed a machine learning model that used the urinary sphingolipids as variables. That model demonstrated high precision and possible usefulness for the differentiation of kidney diseases.

In the pathogenesis of kidney diseases, alterations in urinary sphingolipids might reflect changes in renal function and the associated underlying pathologic processes. The kidneys play a crucial role in sphingolipid metabolism, including synthesis, degradation, and reabsorption. Disruptions in those processes can lead to abnormal accumulation or depletion of sphingolipids, which might further contribute to renal dysfunction (30, 42, 43).

One possible explanation for the associations between the changes of CERs, sphingoid bases, and their phosphates and kidney diseases is the disruption of the sphingolipid signaling pathways observed in those diseases. Sphingolipids are bioactive signaling molecules that regulate cellular processes including inflammation and apoptosis (15, 16). Aberrant sphingolipid signaling can therefore contribute to renal inflammation, fibrosis, and cell death, all of which are characteristic features of kidney diseases. Because inflammation and renal injury can lead to the alteration of sphingolipids, another possible explanation is that the pathologic state in kidney disease might result in the changes of sphingolipid metabolism, as shown in rodent models. Increased levels of kidney cortex CERs or an association between renal apoptosis and CER levels is observed in cisplatin administration (44), unilateral ureteral obstruction (45), and ischemia/reperfusion injury (29).

The observed positive correlation between urinary S1P and OFBs in urine sediment suggests a potential association between urinary S1P and DM. OFBs are lipid-containing components observed in conditions characterized by severe proteinuria, such as nephrotic syndrome, and is listed as a reference in Evidence-Based Clinical Practice Guideline for Nephrotic Syndrome 2017 in Japan. Currently, OFB content has not been completely elucidated, especially with respect to the sphingolipids, including S1P. Further analysis of the lipids contained in OFBs is required to clarify the association between the presence of OFBs in urine and urinary S1P metabolism.

The changes we observed in urinary S1P and dhS1P were different depending on the renal disease, while they were not significantly different depending on GFR stage. Urinary S1P was positively correlated with U-TP, but dhS1P was not similarly correlated. Although these lipid mediators are known to be similarly agonistic to S1P receptors, they reportedly have distinct functions, including differences in their ability to bind to apolipoprotein M or albumin (46). Changes in urinary S1P and dhS1P levels might be influenced by alterations in urinary albumin and/or apolipoprotein M related to kidney disease. Given that the other sphingolipids were positively correlated with U-TP, they might be being leaked from the glomerulus, binding to albumin. In the patients with CGN or SLE, certain specific CERs, such as the CER C24:0, were higher than they were in other groups. In addition, the ratios of very-long chain to long chain ceramide including C22:0/C20:0 and C24:0/C20:0 were higher in the CGN and SLE groups than in the other groups (**Table 1**). Those findings suggest that elevated levels of the CER C24:0 might be associated with the severity of glomerular inflammation. On the other hand, the ratios of C16:0/C24:0 and C18:0/C24:0, previously associated with predicting cardiovascular death (22), were found to be higher in the DM and AH groups compared to the control participants (**Table 1**). While data on cardiovascular complications were not collected in this study, these findings may be relevant, especially considering that patients with DM are often susceptible to cardiovascular events. Further investigation is required to understand the biologic relevance and specific roles of individual CER species. Correlations similar to those in our previous reports (38, 40) between the urinary sphingolipids and U-TP or eGFR were observed in the present study. However, the strength of those correlations might depend on the types of renal diseases or the underlying pathologic conditions. In our previous studies, the CER C24:0 was most strongly correlated with U-TP in patients with DM (38), whereas the CER C16:0 was most strongly correlated with the acute phase in patients hospitalized with COVID-19 (40). Additionally, since the levels of U-TP varied significantly among the kidney disease groups in our present study, we conducted a stratified analysis for the measured urinary sphingolipids, adjusting for the degree of proteinuria (**Supplementary Table S5**). In the group with normal proteinuria, urinary sphingosine, CER C16:0, C18:0, and C18:1 were found to be significantly higher in the SLE group compared to the control group. Meanwhile, in the group with severe proteinuria, only urinary S1P in the DM group and urinary Sph in the SLE group were elevated compared to the other groups. Despite normal proteinuria, changes in the measured urinary sphingolipid levels were observed among the kidney diseases. Therefore, it is suggested that assessing urinary sphingolipids may be valuable in evaluating the pathological condition of kidney injury.

In regard to the clinical usefulness of measuring urinary CERs, sphingoid bases, and their phosphates, our study suggests that these molecules might serve as biomarkers for renal diseases. The ROC analyses demonstrated that the measurement of CERs species, S1P, and their ratios in urine, including the CER C24:0, the CER C22:0, the CER C20:0 to C18:0 ratio, the CER C22:0 to C20:0 ratio, and the CER C24:0 to C22:0, can potentially provide valuable diagnostic information for several diseases (**Figure 1**; **Supplementary Table S3**). However, the urinary CERs, sphingoid bases, and their phosphates were significantly changed in all the kidney disease groups, with the data being widely distributed and overlapped, suggesting that a single urinary sphingolipid might not be a useful marker for differentiating kidney diseases. To overcome that weakness, we constructed a machine learning model based on the test results for the CERs, sphingoid bases, and their phosphates. Machine learning has advanced significantly in recent years and can provide valuable insights when large quantities of data are at issue (47). We aimed to construct a machine learning model that would discriminate kidney diseases, successfully improving the diagnostic accuracy of the standard clinical laboratory data by incorporating urinary sphingolipid data. Nevertheless, an analysis of the importance of the variables in the model suggested that the clinical laboratory data contribute more to the diagnosis than do the urinary sphingolipid measurements (**Figure 3**).

A limitation of our study is that it was retrospective and observational. Moreover, the diagnoses were made in some patients based solely on clinical data rather than biopsy. In addition, the enrolled patients were not receiving identical treatments for their disease. Furthermore, although we could not address the renal prognosis for the patients with targeting diseases by the urinary sphingolipid levels measured in the present study, Hilvo et al. have been reported that the ratio of ceramide predicted incidence of diabetes (48). Therefore, urinary sphingolipids might be a useful biomarker predicting the prognosis of renal dysfunction. Future studies examining larger numbers of patients in time series data with histological findings and independent samples will be necessary to validate the usefulness of the measuring urinary CERs, sphingoid bases, and their phosphates and our machine learning model. Nevertheless, measurement of urinary sphingolipids may support the diagnosis of kidney diseases in a noninvasive manner.

To summarize, urinary CERs, sphingoid bases, and their phosphates show alterations among kidney diseases, suggesting their potential involvement in the development of kidney injury.

## Ethics approval and consent to participate

The study was performed in accordance with the Declaration of Helsinki. Written informed consent for sample analysis was obtained from some patients. For patients whose written informed consent could not be obtained because of discharge or transfer away from the hospital, consent was obtained in the form of an opt-out on a website. Specifically, patients were informed about the study on the website, and those who did not wish to be enrolled in the study were excluded. The study design was approved by The University of Tokyo Medical Research Center Ethics Committee, and because archived specimens were used and all relevant data were retrieved from medical records, the committee members waived the need for written informed consent when written informed consent had not been able to be obtained (nos. 11206, 10266, 2602).

## Data availability statement

The raw data supporting the conclusions of this article will be made available by the authors, without undue reservation.

## Ethics statement

The studies involving humans were approved by The University of Tokyo Medical Research Center Ethics Committee. The studies were conducted in accordance with the local legislation and institutional requirements. Written informed consent for participation was not required from the participants or the participants’ legal guardians/next of kin because some participants discharged or transferred away from the hospital, consent was obtained in the form of an opt-out on a website. Specifically, patients were informed about the study on the website, and those who did not wish to be enrolled in the study were excluded.

## Author contributions

YM: Conceptualization, Data curation, Formal analysis, Funding acquisition, Investigation, Writing – original draft. ES: Data curation, Writing – review & editing. YO: Writing – review & editing. YY: Supervision, Writing – review & editing. MK: Conceptualization, Supervision, Writing – review & editing. HI: Writing – review & editing.
