# Supplementary material for: Alterations in urinary ceramides, sphingoid bases, and their phosphates among patients with kidney disease
Source: Front Nephrol. 2024 Mar 7;4:1343181. doi: 10.3389/fneph.2024.1343181 (PMC10949895; doi:10.3389/fneph.2024.1343181)
Supplement: Supplementary file 1 [file DataSheet_1.pdf]

Figure S1

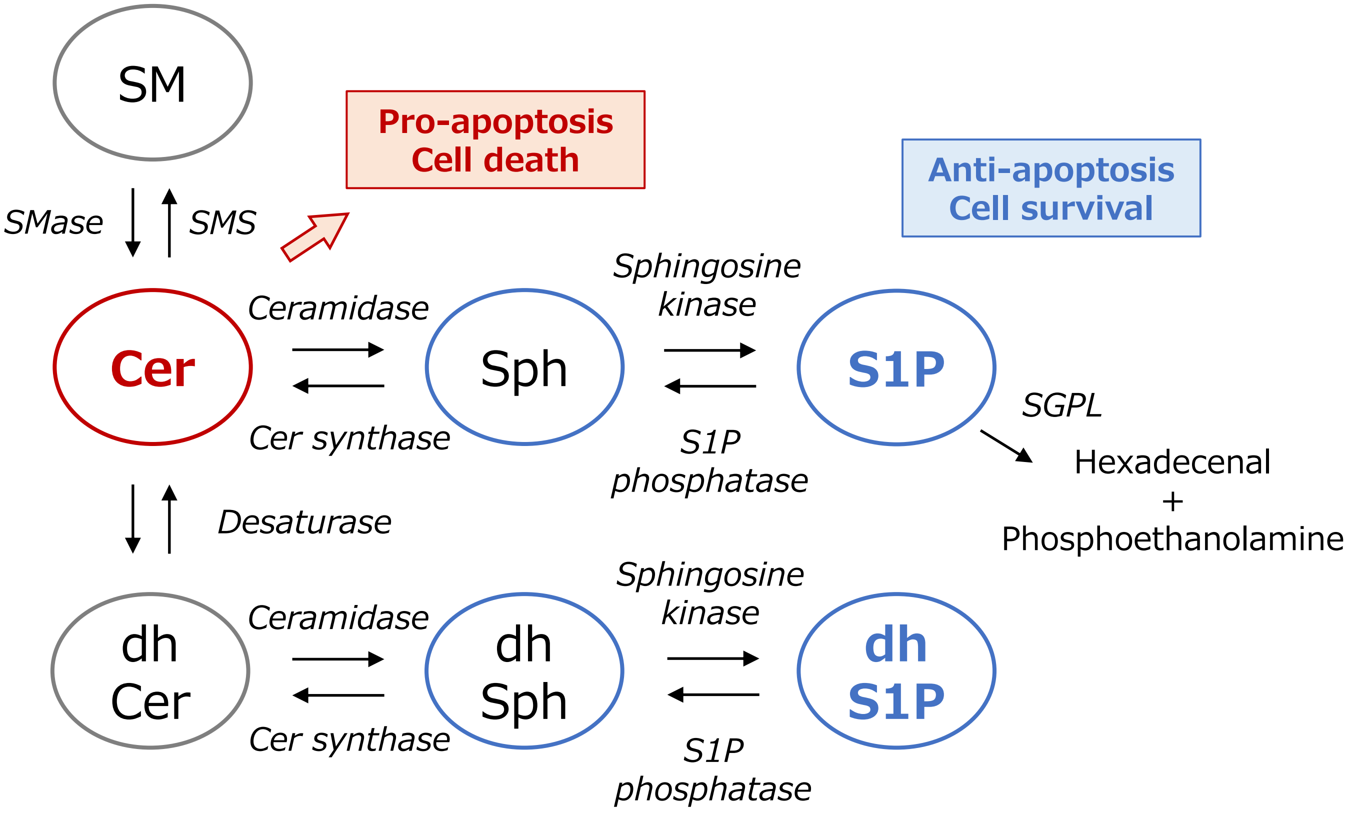

Figure S1. Schema of the sphingolipid metabolism. Ceramide is involved in apoptosis, inflammation, and cell death, while S1P and dhS1P possess roles in anti-apoptosis and cell survival. Cer; ceramide, S1P; sphingosine-1-phosphate, Sph; sphingosine, dhSph; dihydrosphingosine, SPHK; sphingosine kinase, SGPL; S1P lyase, SM; sphingomyelin, SMase; sphingomyelinase, SMS; sphingomyelin synthase

Supplemental Figure S2

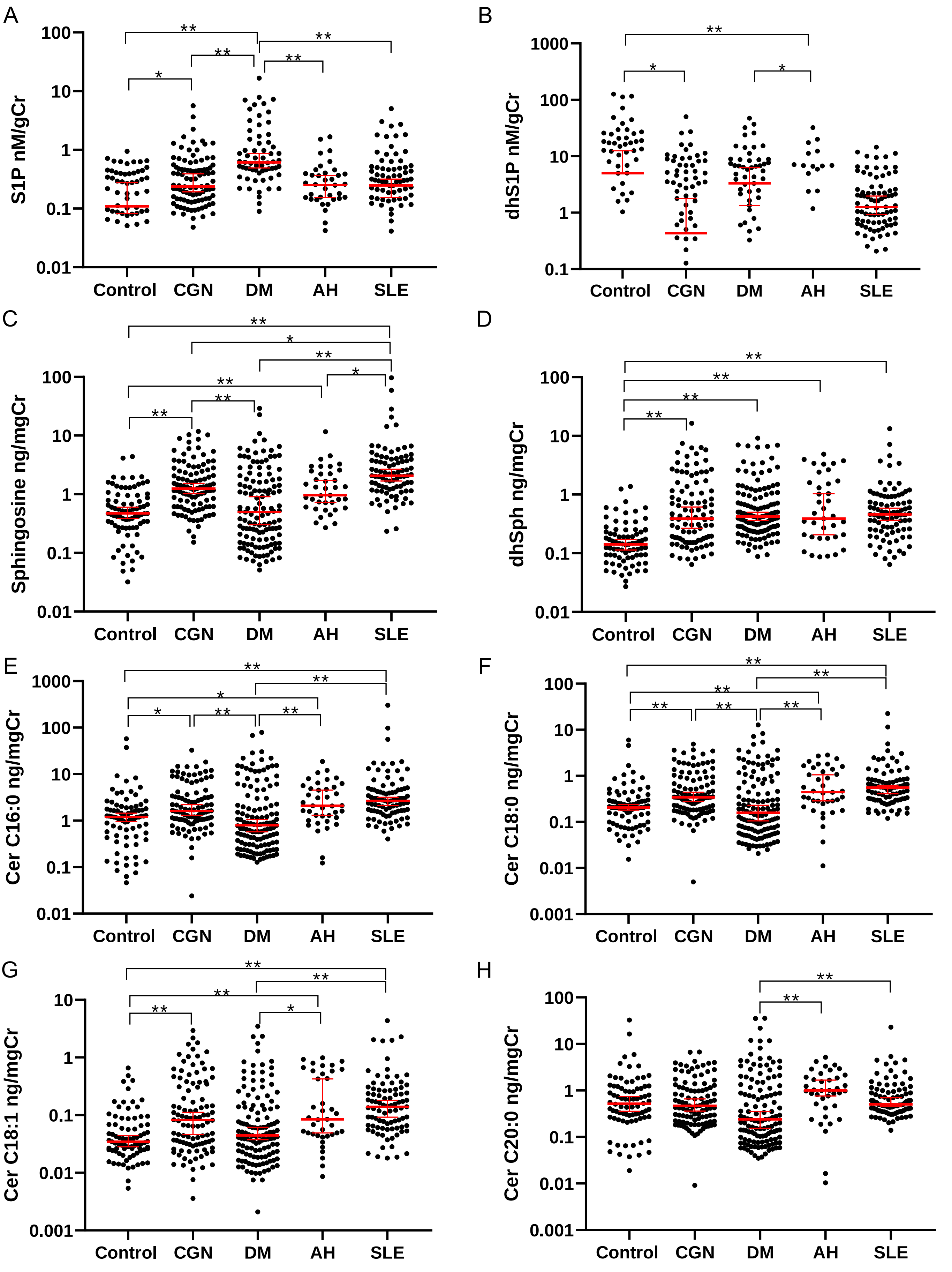

Supplemental Figure S2 (continued)

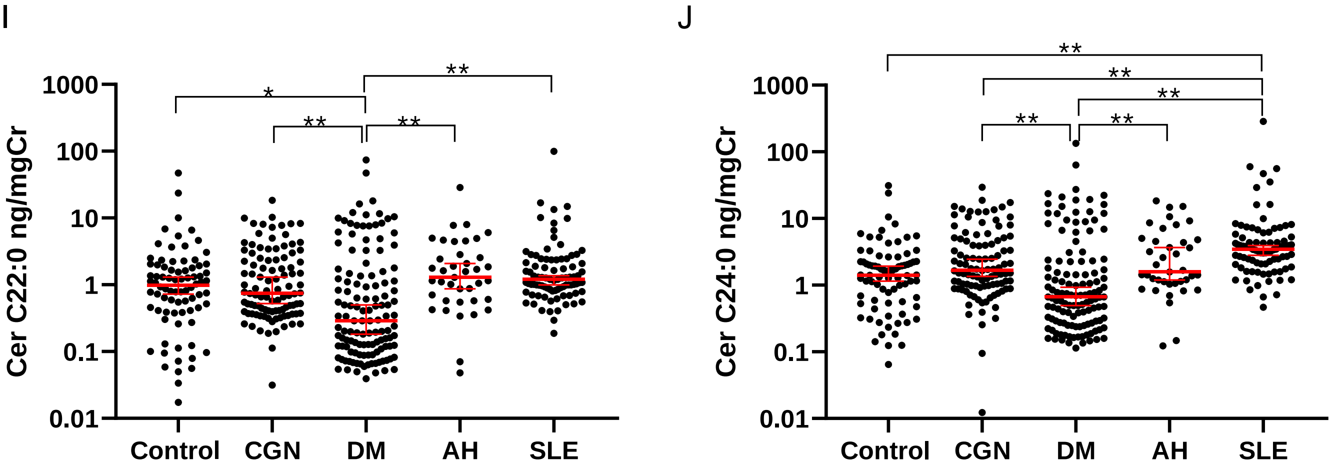

K

	S1P	dhS1P	Sph	dhSph	CER C16:0	CER C18:0	CER C18:1	CER C20:0	CER C22:0	CER C24:0	RBC	renal tubular cells	OFB	hyaline casts	granular casts	epithelial casts	vacuolar denatured casts	fatty casts	RBC casts	WBC casts	waxy casts	eGFR	U-TP
S1P	1.00	0.42	0.36	0.37	0.18	0.29	0.20	0.28	0.19	0.15	0.20	0.13	0.38	0.31	0.31	0.31	0.14	0.34	0.01	0.12	0.27	-0.25	0.54
dhS1P	0.42	1.00	0.05	0.09	-0.22	-0.08	-0.06	0.05	-0.10	-0.14	-0.12	-0.10		-0.16	-0.09	-0.06			-0.10	-0.09	0.01	0.05	0.01
Sph	0.36	0.05	1.00	0.69	0.71	0.78	0.78	0.61	0.64	0.69	0.21	0.18	0.34	0.23	0.33	0.23	0.10	0.26	0.03	0.13	0.24	-0.21	0.47
dhSph	0.37	0.09	0.69	1.00	0.44	0.54	0.70	0.40	0.38	0.42	0.14	0.11	0.27	0.25	0.27	0.18	0.11	0.20		0.06	0.19	-0.22	0.46
CER C16:0	0.18	-0.22	0.71	0.44	1.00	0.94	0.74	0.87	0.92	0.92	0.16	0.18	0.33	0.25	0.26	0.20	0.10	0.25	0.02	0.16	0.24	-0.25	0.40
CER C18:0	0.29	-0.08	0.78	0.54	0.94	1.00	0.85	0.91	0.92	0.92	0.17	0.15	0.35	0.23	0.25	0.19	0.08	0.26	0.01	0.15	0.21	-0.27	0.44
CER C18:1	0.20	-0.06	0.78	0.70	0.74	0.85	1.00	0.68	0.69	0.74	0.10	0.04	0.25	0.14	0.17	0.09	0.07	0.17	-0.05	0.08	0.16	-0.20	0.41
CER C20:0	0.28	0.05	0.61	0.40	0.87	0.91	0.68	1.00	0.95	0.87	0.06	0.11	0.33	0.19	0.22	0.16	0.08	0.25	-0.03	0.16	0.20	-0.26	0.30
CER C22:0	0.19	-0.10	0.64	0.38	0.92	0.92	0.69	0.95	1.00	0.95	0.12	0.16	0.35	0.20	0.24	0.19	0.09	0.27	0.02	0.16	0.23	-0.21	0.31
CER C24:0	0.15	-0.14	0.69	0.42	0.92	0.92	0.74	0.87	0.95	1.00	0.19	0.17	0.34	0.17	0.22	0.17	0.06	0.25	0.05	0.12	0.20	-0.17	0.34
RBC	0.20	-0.12	0.21	0.14	0.16	0.17	0.10	0.06	0.12	0.19	1.00	0.31	0.32	0.33	0.33	0.40	-0.03	0.30	0.50	0.21	0.13	-0.05	0.35
renal tubular cells	0.13	-0.10	0.18	0.11	0.18	0.15	0.04	0.11	0.16	0.17	0.31	1.00	0.36	0.47	0.48	0.51	0.12	0.37	0.32	0.17	0.28	-0.17	0.31
OFB	0.38		0.34	0.27	0.33	0.35	0.25	0.33	0.35	0.34	0.32	0.36	1.00	0.51	0.59	0.52	0.25	0.80	0.17	0.25	0.49	-0.35	0.59
hyaline casts	0.31	-0.16	0.23	0.25	0.25	0.23	0.14	0.19	0.20	0.17	0.33	0.47	0.51	1.00	0.63	0.66	0.21	0.54	0.26	0.24	0.37	-0.52	0.65
granular casts	0.31	-0.09	0.33	0.27	0.26	0.25	0.17	0.22	0.24	0.22	0.33	0.48	0.59	0.63	1.00	0.69	0.35	0.58	0.35	0.29	0.58	-0.42	0.58
epithelial casts	0.31	-0.06	0.23	0.18	0.20	0.19	0.09	0.16	0.19	0.17	0.40	0.51	0.52	0.66	0.69	1.00	0.26	0.59	0.46	0.28	0.47	-0.34	0.58
vacuolar denatured casts	0.14		0.10	0.11	0.10	0.08	0.07	0.08	0.09	0.06	-0.03	0.12	0.25	0.21	0.35	0.26	1.00	0.26	-0.06	0.11	0.47	-0.24	0.25
fatty casts	0.34		0.26	0.20	0.25	0.26	0.17	0.25	0.27	0.25	0.30	0.37	0.80	0.54	0.58	0.59	0.26	1.00	0.20	0.30	0.45	-0.31	0.57
RBC casts	0.01	-0.10	0.03		0.02	0.01	-0.05	-0.03	0.02	0.05	0.50	0.32	0.17	0.26	0.35	0.46	-0.06	0.20	1.00	0.26	0.11	-0.05	0.15
WBC casts	0.12	-0.09	0.13	0.06	0.16	0.15	0.08	0.16	0.16	0.12	0.21	0.17	0.25	0.24	0.29	0.28	0.11	0.30	0.26	1.00	0.25	-0.11	0.19
waxy casts	0.27	0.01	0.24	0.19	0.24	0.21	0.16	0.20	0.23	0.20	0.13	0.28	0.49	0.37	0.58	0.47	0.47	0.45	0.11	0.25	1.00	-0.32	0.40
eGFR	-0.25	0.05	-0.21	-0.22	-0.25	-0.27	-0.20	-0.26	-0.21	-0.17	-0.05	-0.17	-0.35	-0.52	-0.42	-0.34	-0.24	-0.31	-0.05	-0.11	-0.32	1.00	-0.55
U-TP	0.54	0.01	0.47	0.46	0.40	0.44	0.41	0.30	0.31	0.34	0.35	0.31	0.59	0.65	0.58	0.58	0.25	0.57	0.15	0.19	0.40	-0.55	1.00

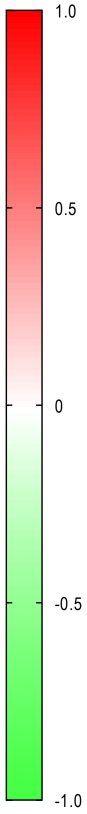

Figure S2. Alteration of the urinary sphingolipids among kidney disease. Levels of the urinary sphingolipids (A) sphingosine 1-phosphate (S1P), (B) dihydroS1P (dhS1P), (C) sphingosine, (D) dihydrosphingosine (dhSph), and the ceramides (E) C16:0, (F) C18:0, (G) C18:1, (H) C20:0, (I) C22:0, and (J) C24:0 by kidney disease type—chronic glomerulonephritis (CGN), diabetes mellitus (DM), arterial hypertension (AH), and systemic lupus erythematosus (SLE)—are shown. The red bar indicates the median ± the 95% confidence interval. **P* < 0.05, ***P* < 0.01. (K) Correlation matrix for the urinary sphingolipid measurements and the urinary sediment results is shown. Positive correlations are indicated by a red gradient, and negative correlations are indicated by a green gradient.
