# Supplementary material for: Alterations in urinary ceramides, sphingoid bases, and their phosphates among patients with kidney disease
Source: Front Nephrol. 2024 Mar 7;4:1343181. doi: 10.3389/fneph.2024.1343181 (PMC10949895; doi:10.3389/fneph.2024.1343181)
Supplement: Supplementary file 2 [file Table_1.pdf]

Supplemental Data

Alterations in urinary ceramides, sphingoid bases, and their phosphates among patients with kidney disease

The Frontiers in Nephrology

Yoshifumi Morita,[1] Eri Sakai,[1] Hideaki Isago,[1,2] Yoshikazu Ono,[1] Yutaka Yatomi,[1,2] Makoto Kurano[1,2]

[1]Department of Clinical Laboratory, The University of Tokyo Hospital, Tokyo, Japan

[2]Department of Clinical Laboratory Medicine, Graduate School of Medicine, The University of Tokyo, Tokyo, Japan

Corresponding author:

Yoshifumi Morita, PhD, and Makoto Kurano, MD, PhD

Department of Clinical Laboratory, University of Tokyo Hospital,

The University of Tokyo, 7-3-1 Hongo, Bunkyo-ku, Tokyo 113-8655, Japan

Tel: +81-3-3815-5411; Fax: +81-3-5689-0495

E-mail: moritay-lab@h.u-tokyo.ac.jp (Y.M.) and kurano-tky@umin.ac.jp (M.K.)

Supplemental Tables

Table S1. Clinical characteristics of participants

		control	chronic glomerulonephritis	diabetes mellitus	arterial hypertension	Systemic lupus nephritis	*P* value
n		80	126	167	39	88	
Urinary chemistry (dipstick)							
SG		1.02 [1.01, 1.03]	1.01 [1.00, 1.03]	1.02 [1.00, 1.03]	1.01 [1.01, 1.03]	1.01 [1.00, 1.03]	0.001
pH (%)							
	5.0	12 (15.0)	44 (34.9)	53 (31.7)	16 (41.0)	13 (17.3)	0.012
	5.5	21 (26.2)	23 (18.3)	44 (26.3)	12 (30.8)	14 (18.7)	
	6.0	17 (21.2)	24 (19.0)	19 (11.4)	4 (10.3)	15 (20.0)	
	6.5	20 (25.0)	16 (12.7)	20 (12.0)	3 (7.7)	14 (18.7)	
	7.0	5 (6.2)	17 (13.5)	19 (11.4)	3 (7.7)	13 (17.3)	
	7.5	4 (5.0)	2 (1.6)	8 (4.8)	1 (2.6)	4 (5.3)	
	8.0	0 (0.0)	0 (0.0)	3 (1.8)	0 (0.0)	2 (2.7)	
	8.5	1 (1.2)	0 (0.0)	1 (0.6)	0 (0.0)	0 (0.0)	
BLD							
	(-)	80 (100.0)	41 (32.5)	127 (76.0)	34 (87.2)	57 (76.0)	<0.001
	(1+)	0 (0.0)	12 (9.5)	25 (15.0)	2 (5.1)	5 (6.7)	
	(2+)	0 (0.0)	30 (23.8)	14 (8.4)	3 (7.7)	6 (8.0)	
	(3+)	0 (0.0)	43 (34.1)	1 (0.6)	0 (0.0)	7 (9.3)	
GLU							
	(-)	80 (100.0)	125 (99.2)	102 (61.1)	38 (97.4)	70 (93.3)	<0.001
	(1+)	0 (0.0)	0 (0.0)	11 (6.6)	0 (0.0)	4 (5.3)	
	(2+)	0 (0.0)	0 (0.0)	12 (7.2)	0 (0.0)	1 (1.3)	
	(3+)	0 (0.0)	0 (0.0)	19 (11.4)	0 (0.0)	0 (0.0)	
	(4+)	0 (0.0)	1 (0.8)	23 (13.8)	1 (2.6)	0 (0.0)	
Urinary sediment							
RBC (%)	<1 /HPF	69 (86.2)	29 (23.6)	105 (64.8)	28 (71.8)	45 (51.7)	<0.001
	1-4 /HPF	10 (12.5)	21 (17.1)	39 (24.1)	9 (23.1)	20 (23.0)	
	5-9 /HPF	1 (1.2)	13 (10.6)	11 (6.8)	2 (5.1)	4 (4.6)	
	10-19 /HPF	0 (0.0)	17 (13.8)	4 (2.5)	0 (0.0)	7 (8.0)	
	20-29 /HPF	0 (0.0)	13 (10.6)	3 (1.9)	0 (0.0)	3 (3.4)	
	30-49 /HPF	0 (0.0)	11 (8.9)	0 (0.0)	0 (0.0)	3 (3.4)	
	50-99 /HPF	0 (0.0)	11 (8.9)	0 (0.0)	0 (0.0)	2 (2.3)	
	100≤ /HPF	0 (0.0)	8 (6.5)	0 (0.0)	0 (0.0)	3 (3.4)	

WBC (%)	<1 /HPF	55 (68.8)	54 (43.9)	104 (64.2)	33 (84.6)	33 (37.9)	<0.001
	1-4 /HPF	23 (28.7)	45 (36.6)	34 (21.0)	5 (12.8)	31 (35.6)	
	5-9 /HPF	1 (1.2)	15 (12.2)	12 (7.4)	0 (0.0)	9 (10.3)	
	10-19 /HPF	1 (1.2)	6 (4.9)	5 (3.1)	0 (0.0)	6 (6.9)	
	20-29 /HPF	0 (0.0)	1 (0.8)	3 (1.9)	0 (0.0)	2 (2.3)	
	30-49 /HPF	0 (0.0)	2 (1.6)	0 (0.0)	0 (0.0)	3 (3.4)	
	50-99 /HPF	0 (0.0)	0 (0.0)	1 (0.6)	1 (2.6)	1 (1.1)	
	100≤ /HPF	0 (0.0)	0 (0.0)	3 (1.9)	0 (0.0)	2 (2.3)	
Renal tubular cells (%)	(-)	80 (100.0)	89 (72.4)	141 (87.0)	34 (87.2)	69 (84.1)	<0.001
	<1 /HPF	0 (0.0)	20 (16.3)	13 (8.0)	4 (10.3)	9 (11.0)	
	1≤ /HPF	0 (0.0)	14 (11.4)	8 (4.9)	1 (2.6)	4 (4.9)	
Oval fat bodies (%)	(-)	80 (100.0)	82 (66.7)	131 (80.9)	31 (79.5)	69 (84.1)	0.002
	1-4 /WF	0 (0.0)	11 (8.9)	13 (8.0)	4 (10.3)	3 (3.7)	
	5-9 /WF	0 (0.0)	9 (7.3)	7 (4.3)	2 (5.1)	2 (2.4)	
	10-19 /WF	0 (0.0)	10 (8.1)	3 (1.9)	1 (2.6)	2 (2.4)	
	20-29 /WF	0 (0.0)	3 (2.4)	1 (0.6)	0 (0.0)	2 (2.4)	
	30-49 /WF	0 (0.0)	1 (0.8)	4 (2.5)	1 (2.6)	2 (2.4)	
	50-99 /WF	0 (0.0)	4 (3.3)	3 (1.9)	0 (0.0)	2 (2.4)	
	100≤ /WF	0 (0.0)	3 (2.4)	0 (0.0)	0 (0.0)	0 (0.0)	
Hyaline casts (%)	(-)	63 (78.8)	15 (12.2)	45 (27.8)	7 (17.9)	46 (56.1)	<0.001
	1-4 /WF	17 (21.2)	10 (8.1)	25 (15.4)	2 (5.1)	6 (7.3)	
	5-9 /WF	0 (0.0)	17 (13.8)	10 (6.2)	8 (20.5)	3 (3.7)	
	10-19 /WF	0 (0.0)	22 (17.9)	19 (11.7)	5 (12.8)	3 (3.7)	
	20-29 /WF	0 (0.0)	11 (8.9)	16 (9.9)	4 (10.3)	2 (2.4)	
	30-49 /WF	0 (0.0)	13 (10.6)	11 (6.8)	4 (10.3)	6 (7.3)	
	50-99 /WF	0 (0.0)	18 (14.6)	11 (6.8)	1 (2.6)	6 (7.3)	
	100≤ /WF	0 (0.0)	17 (13.8)	25 (15.4)	8 (20.5)	10 (12.2)	
Epithelial casts (%)	(-)	80 (100.0)	51 (41.5)	109 (67.3)	30 (76.9)	61 (74.4)	<0.001
	1-4 /WF	0 (0.0)	36 (29.3)	36 (22.2)	6 (15.4)	15 (18.3)	
	5-9 /WF	0 (0.0)	19 (15.4)	8 (4.9)	1 (2.6)	3 (3.7)	
	10-19 /WF	0 (0.0)	11 (8.9)	7 (4.3)	2 (5.1)	1 (1.2)	
	20-29 /WF	0 (0.0)	4 (3.3)	1 (0.6)	0 (0.0)	2 (2.4)	
	30-49 /WF	0 (0.0)	2 (1.6)	1 (0.6)	0 (0.0)	0 (0.0)	
Granular casts (%)	(-)	80 (100.0)	67 (54.5)	113 (69.8)	29 (74.4)	66 (80.5)	<0.001
	1-4 /WF	0 (0.0)	23 (18.7)	19 (11.7)	5 (12.8)	7 (8.5)	
	5-9 /WF	0 (0.0)	15 (12.2)	7 (4.3)	1 (2.6)	4 (4.9)	

	10-19 /WF	0 (0.0)	9 (7.3)	10 (6.2)	2 (5.1)	3 (3.7)	
	20-29 /WF	0 (0.0)	5 (4.1)	2 (1.2)	0 (0.0)	1 (1.2)	
	30-49 /WF	0 (0.0)	1 (0.8)	3 (1.9)	1 (2.6)	0 (0.0)	
	50-99 /WF	0 (0.0)	3 (2.4)	3 (1.9)	1 (2.6)	1 (1.2)	
	100≤ /WF	0 (0.0)	0 (0.0)	5 (3.1)	0 (0.0)	0 (0.0)	
Waxy casts (%)	(-)	80 (100.0)	109 (88.6)	142 (87.7)	37 (94.9)	76 (92.7)	0.269
	1-4 /WF	0 (0.0)	13 (10.6)	12 (7.4)	1 (2.6)	4 (4.9)	
	5-9 /WF	0 (0.0)	0 (0.0)	4 (2.5)	1 (2.6)	1 (1.2)	
	10-19 /WF	0 (0.0)	0 (0.0)	1 (0.6)	0 (0.0)	1 (1.2)	
	20-29 /WF	0 (0.0)	1 (0.8)	0 (0.0)	0 (0.0)	0 (0.0)	
	30-49 /WF	0 (0.0)	0 (0.0)	2 (1.2)	0 (0.0)	0 (0.0)	
	50-99 /WF	0 (0.0)	0 (0.0)	0 (0.0)	0 (0.0)	0 (0.0)	
	100≤ /WF	0 (0.0)	0 (0.0)	1 (0.6)	0 (0.0)	0 (0.0)	
Fatty casts (%)	(-)	80 (100.0)	82 (66.7)	131 (80.9)	31 (79.5)	68 (82.9)	0.009
	1-4 /WF	0 (0.0)	23 (18.7)	17 (10.5)	7 (17.9)	7 (8.5)	
	5-9 /WF	0 (0.0)	7 (5.7)	7 (4.3)	0 (0.0)	4 (4.9)	
	10-19 /WF	0 (0.0)	7 (5.7)	5 (3.1)	1 (2.6)	2 (2.4)	
	20-29 /WF	0 (0.0)	0 (0.0)	1 (0.6)	0 (0.0)	1 (1.2)	
	30-49 /WF	0 (0.0)	1 (0.8)	1 (0.6)	0 (0.0)	0 (0.0)	
	50-99 /WF	0 (0.0)	2 (1.6)	0 (0.0)	0 (0.0)	0 (0.0)	
	100≤ /WF	0 (0.0)	1 (0.8)	0 (0.0)	0 (0.0)	0 (0.0)	
RBC casts (%)	(-)	80 (100.0)	85 (69.1)	159 (98.1)	39 (100.0)	75 (91.5)	<0.001
	1-4 /WF	0 (0.0)	31 (25.2)	3 (1.9)	0 (0.0)	6 (7.3)	
	5-9 /WF	0 (0.0)	6 (4.9)	0 (0.0)	0 (0.0)	1 (1.2)	
	10-19 /WF	0 (0.0)	1 (0.8)	0 (0.0)	0 (0.0)	0 (0.0)	
WBC casts (%)	(-)	80 (100.0)	113 (91.9)	160 (98.8)	38 (97.4)	80 (97.6)	0.018
	1-4 /WF	0 (0.0)	8 (6.5)	1 (0.6)	1 (2.6)	0 (0.0)	
	5-9 /WF	0 (0.0)	2 (1.6)	1 (0.6)	0 (0.0)	1 (1.2)	
	10-19 /WF	0 (0.0)	0 (0.0)	0 (0.0)	0 (0.0)	0 (0.0)	
	20-29 /WF	0 (0.0)	0 (0.0)	0 (0.0)	0 (0.0)	1 (1.2)	
Vacuolar denatured casts (%)	(-)	80 (100.0)	119 (96.7)	153 (94.4)	37 (94.9)	82 (100.0)	0.514
	1-4 /WF	0 (0.0)	3 (2.4)	6 (3.7)	2 (5.1)	0 (0.0)	
	5-9 /WF	0 (0.0)	1 (0.8)	2 (1.2)	0 (0.0)	0 (0.0)	
	10-19 /WF	0 (0.0)	0 (0.0)	0 (0.0)	0 (0.0)	0 (0.0)	
	20-29 /WF	0 (0.0)	0 (0.0)	0 (0.0)	0 (0.0)	0 (0.0)	
	30-49 /WF	0 (0.0)	0 (0.0)	0 (0.0)	0 (0.0)	0 (0.0)	

50-99 /WF	0 (0.0)	0 (0.0)	0 (0.0)	0 (0.0)	0 (0.0)
100≤ /WF	0 (0.0)	0 (0.0)	1 (0.6)	0 (0.0)	0 (0.0)

Data was presented as median [range] for SG.
Abbreviations: HPF, high power field; RBC, red blood cells; SG, specific gravity; WBC, white blood cells; WF, whole field.

Table S2. Correlation analysis between urinary sphingolipids and eGFR and urinary total protein by kidney disease group

	Chronic glomerulonephritis		Diabetes mellitus		Arterial hypertension		Systemic lupus nephritis	
urinary total protein	r_s	*P* value	r_s	*P* value	r_s	*P* value	r_s	*P* value
sphingosine 1-phosphate	0.47	<0.001	0.68	<0.001	0.42	0.007	0.41	<0.001
dihydrosphingosine 1-phosphate	0.16	0.101	0.06	0.669	-0.03	0.839	0.34	0.001
sphingosine	0.44	<0.001	0.71	<0.001	0.24	0.137	0.36	0.001
dihydrosphingosine	0.30	0.002	0.40	<0.001	0.09	0.595	0.42	<0.001
ceramide C16:0	0.36	<0.001	0.69	<0.001	0.13	0.415	0.53	<0.001
ceramide C18:0	0.38	<0.001	0.69	<0.001	0.16	0.316	0.58	<0.001
ceramide C18:1	0.25	0.010	0.62	<0.001	0.20	0.215	0.46	<0.001
ceramide C20:0	0.33	0.001	0.66	<0.001	-0.02	0.903	0.51	<0.001
ceramide C22:0	0.40	<0.001	0.71	<0.001	0.10	0.546	0.57	<0.001
ceramide C24:0	0.39	<0.001	0.69	<0.001	0.17	0.294	0.57	<0.001
eGFR								
sphingosine 1-phosphate	0.03	0.743	-0.17	0.191	-0.40	0.014	-0.17	0.116
dihydrosphingosine 1-phosphate	-0.08	0.403	-0.05	0.720	-0.28	0.091	0.06	0.572
sphingosine	-0.17	0.089	-0.51	<0.001	-0.20	0.220	0.14	0.209
dihydrosphingosine	-0.18	0.071	-0.19	0.031	0.00	0.987	0.08	0.456
ceramide C16:0	-0.21	0.034	-0.48	<0.001	-0.16	0.338	-0.07	0.496
ceramide C18:0	-0.21	0.032	-0.50	<0.001	-0.25	0.127	-0.06	0.586
ceramide C18:1	-0.14	0.152	-0.43	<0.001	-0.20	0.238	0.08	0.454
ceramide C20:0	-0.19	0.050	-0.49	<0.001	-0.17	0.296	-0.10	0.362
ceramide C22:0	-0.18	0.070	-0.50	<0.001	-0.18	0.268	-0.16	0.138
ceramide C24:0	-0.13	0.197	-0.46	<0.001	-0.19	0.242	-0.07	0.504

Table S3. ROC analysis

	CGN versus DM			CGN versus arterial hypertension			CGN versus SLE		
	variables	AUC	*P* value	variables	AUC	*P* value	variables	AUC	*P* value
1	RBC	0.776	2.008E-18	RBC	0.798	3.805E-08	Cer C24:0/TP	0.816	2.154E-12
2	BLD	0.770	1.196E-20	BLD	0.798	1.169E-10	PRO	0.787	1.428E-17
3	dysmorphic RBC	0.758	1.198E-24	dysmorphic RBC	0.774	7.033E-10	Cer C22:0/TP	0.786	1.015E-12
4	age	0.707	2.436E-11	age	0.768	2.438E-06	Cer C16:0/TP	0.783	7.77E-13
5	GLU	0.690	3.201E-15	C20:0/C18:0	0.721	8.7E-05	Cer C18:0/TP	0.780	3.694E-12
6	S1P/C20:0	0.684	2.193E-10	gender	0.708	1.908E-06	Cer C18:1/TP	0.761	4.005E-11
7	C22:0/C20:0	0.675	0.0037964	Cer C20:0	0.701	0.0005321	Cer C20:0/TP	0.750	1.537E-10
8	S1P/C16:0	0.672	6.879E-09	Cer C20:0/TP	0.695	0.0001031	BLD	0.747	4.911E-13
9	S1P/C22:0	0.672	2.784E-09	WBC	0.692	0.0023275	C24:0/C20:0	0.738	3.609E-08
10	S1P/C18:0	0.663	1.857E-08	Cer C22:0/TP	0.689	0.0001552	C24:0/C22:0	0.725	4.628E-07
	DM versus arterial hypertension			DM versus SLE			arterial hypertension versus SLE		
	variables	AUC	*P* value	variables	AUC	*P* value	variables	AUC	*P* value
1	Cer C22:0/TP	0.770	1.146E-07	C22:0/C20:0	0.886	1.776E-16	gender	0.893	4.806E-24
2	Cer C22:0	0.770	4.838E-07	Cer C24:0/TP	0.880	1.387E-20	age	0.862	3.916E-10
3	C22:0/C16:0	0.769	1.439E-05	C24:0/C20:0	0.875	2.565E-13	C24:0/C22:0	0.851	1.976E-07
4	Cer C24:0	0.764	3.873E-06	Cer C22:0/TP	0.863	4.201E-19	eGFR	0.850	4.054E-09
5	Cer C18:0	0.756	3.752E-06	Cer C18:0/TP	0.846	4.541E-17	C24:0/C20:0	0.799	1.036E-07
6	Cer C20:0	0.754	6.269E-06	Cer C16:0/TP	0.842	3.544E-17	dhSph/Sph	0.782	0.0002444
7	Cer C16:0	0.752	7.644E-06	Cer C24:0	0.823	7.794E-17	C20:0/C16:0	0.751	2.54E-05
8	C22:0/C18:0	0.745	1.571E-05	C24:0/C18:0	0.816	9.535E-10	C20:0/C18:0	0.743	1.673E-05
9	Cer C18:1	0.737	7.205E-06	Sphingosine	0.813	9.788E-18	WBC	0.735	5.236E-05
10	Cer C24:0/TP	0.732	2.335E-06	Cer C18:1/TP	0.812	3.347E-15	C24:0/C18:0	0.719	0.0009374

Abbreviations: AUC, area under curve; CER, ceramide; CGN, chronic glomerulonephritis; dhS1P, dihydrosphingosine 1-phosphate; dhSph, dihydrosphingosine; DM, diabetes mellitus; S1P, sphingosine 1-phosphate; SLE, systemic lupus erythematosus; Sph, sphingosine; TP, total protein.

Table S4. The constructed machine learning models

**the only clinical laboratory data**					
	**Accuracy**	**94.03%**	**Precision**	**66.96%**	
Training	Control	CGN	DM	AH	SLE
Control	58	0	0	0	0
CGN	1	88	1	1	1
DM	6	1	117	1	4
AH	0	0	0	29	1
SLE	0	1	5	0	70
Testing	Control	CGN	DM	AH	SLE
Control	19	0	3	0	0
CGN	0	21	7	0	6
DM	1	4	31	1	1
AH	0	0	7	2	0
SLE	1	4	3	0	4
**the urinary sphingolipids were added**					
	**Accuracy**	**96.10%**	**Precision**	**78.26%**	
Training	Control	CGN	DM	AH	SLE
Control	56	0	1	0	1
CGN	1	91	0	0	0
DM	0	0	126	2	1
AH	0	3	1	23	3
SLE	0	2	0	0	74
Testing	Control	CGN	DM	AH	SLE
Control	21	1	0	0	0
CGN	1	24	2	0	7
DM	0	3	34	0	1
AH	0	3	2	1	3
SLE	0	1	1	0	10

Abbreviations: AH, arterial hypertension; CGN, chronic glomerulonephritis; DM, diabetes mellitus; SLE, systemic lupus erythematosus.

Table S5. Stratified analysis for the urinary ceramides, sphingoid bases, and their phosphates among kidney disease adjusted by degree of proteinuria

	control	chronic glomerulonephritis	diabetes mellitus	arterial hypertension	systemic lupus erytematosus	*P* value
Normal (less than 150 mg/gCr)						
n	80	17	65	10	35	
S1P (nmol/mgCr)	0.11 [0.00, 0.39]	0.13 [0.10, 0.22]	0.45 [0.25, 0.47]	0.20 [0.11, 0.34]	0.19 [0.05, 0.30]	0.178
dhS1P (nmol/mgCr)	5.02 [0.00, 18.24]	0.06 [0.00, 0.89]	4.29 [2.43, 7.47] †	1.21 [0.00, 6.41]	0.91 [0.48, 1.68] ‡	0.022
Sphingosine (ng/mgCr)	0.47 [0.27, 0.95]	0.61 [0.53, 1.01]	0.17 [0.10, 0.30] *, †, ‡	0.82 [0.54, 1.66]	1.40 [0.98, 2.36] *, †, ‡	<0.001
dhSph (ng/mgCr)	0.14 [0.08, 0.23]	0.16 [0.10, 0.18]	0.38 [0.24, 0.57] *, †	0.42 [0.18, 0.77]	0.29 [0.18, 0.48] *,	<0.001
CER C16:0 (ng/mgCr)	1.20 [0.53, 1.89]	1.27 [0.81, 2.05]	0.31 [0.21, 0.57] *, †	2.17 [1.59, 3.61] ‡	1.63 [1.08, 2.57] *, ‡	<0.001
CER C18:0 (ng/mgCr)	0.20 [0.10, 0.30]	0.29 [0.12, 0.34]	0.06 [0.04, 0.11] *, †	0.39 [0.29, 0.94] ‡	0.31 [0.24, 0.44] *, ‡	<0.001
CER C18:1 (ng/mgCr)	0.03 [0.02, 0.07]	0.04 [0.03, 0.11]	0.02 [0.02, 0.04]	0.05 [0.04, 0.34]	0.08 [0.05, 0.13] *, ‡	<0.001
CER C20:0 (ng/mgCr)	0.52 [0.27, 1.14]	0.34 [0.20, 0.60]	0.09 [0.06, 0.17] *, †	1.07 [0.79, 2.03] ‡	0.37 [0.29, 0.49] ‡, §	<0.001
CER C22:0 (ng/mgCr)	0.98 [0.41, 1.87]	0.52 [0.38, 0.74]	0.10 [0.07, 0.19] *, †	1.46 [0.92, 2.03] ‡	0.82 [0.59, 1.03] ‡	<0.001
CER C24:0 (ng/mgCr)	1.42 [0.59, 2.25]	1.04 [0.85, 1.90]	0.28 [0.18, 0.50] *, †	1.84 [1.21, 2.85] ‡	2.09 [1.52, 3.14] †, ‡	<0.001
Moderate (150-499 mg/gCr)						
n	0	32	34	10	22	
S1P (nmol/mgCr)	-	0.22 [0.14, 0.42]	0.51 [0.34, 0.63]	0.16 [0.08, 0.24] ‡	0.14 [0.00, 0.31] ‡	0.005
dhS1P (nmol/mgCr)	-	0.36 [0.00, 4.42]	3.01 [0.00, 6.57]	0.00 [0.00, 5.02]	2.37 [0.93, 4.92]	0.303
Sphingosine (ng/mgCr)	-	1.36 [0.65, 2.09]	0.38 [0.17, 0.57] †	0.85 [0.44, 1.18] ‡	2.09 [1.58, 3.66] §	<0.001
dhSph (ng/mgCr)	-	0.45 [0.23, 1.31]	0.36 [0.18, 0.50]	0.35 [0.12, 1.03]	0.54 [0.36, 0.96]	0.24
CER C16:0 (ng/mgCr)	-	1.19 [0.76, 2.90]	0.71 [0.44, 1.20]	1.68 [1.26, 2.10] ‡	2.89 [1.64, 6.90] †, ‡	<0.001
CER C18:0 (ng/mgCr)	-	0.23 [0.18, 0.60]	0.14 [0.10, 0.23] †	0.31 [0.18, 1.16] ‡	0.56 [0.43, 0.85] †, ‡	<0.001
CER C18:1 (ng/mgCr)	-	0.05 [0.03, 0.35]	0.04 [0.02, 0.06]	0.07 [0.04, 0.36]	0.21 [0.09, 0.30] ‡	0.001
CER C20:0 (ng/mgCr)	-	0.31 [0.19, 0.73]	0.22 [0.13, 0.34]	0.84 [0.35, 1.48] ‡	0.66 [0.42, 1.13] ‡	<0.001
CER C22:0 (ng/mgCr)	-	0.44 [0.31, 2.03]	0.24 [0.13, 0.48] †	0.99 [0.49, 1.61] ‡	1.29 [1.01, 2.41] †, ‡	<0.001
CER C24:0 (ng/mgCr)	-	1.06 [0.73, 3.44]	0.66 [0.40, 0.83] †	1.28 [0.89, 2.77] ‡	3.98 [2.81, 6.22] †, ‡, §	<0.001
Severe (over 500 mg/gCr)						
n	0	77	68	19	31	
S1P (nmol/mgCr)	-	0.37 [0.15, 0.73]	0.87 [0.48, 2.53] †	0.32 [0.17, 0.46] ‡	0.47 [0.26, 0.92] ‡	<0.001
dhS1P (nmol/mgCr)	-	0.00 [0.00, 5.03]	3.23 [0.12, 8.62]	0.00 [0.00, 3.96]	1.66 [0.71, 2.93]	0.012
Sphingosine (ng/mgCr)	-	1.45 [0.79, 2.95]	1.82 [0.93, 4.41]	1.49 [0.73, 2.73]	3.39 [1.78, 5.81] †, §	0.01
dhSph (ng/mgCr)	-	0.50 [0.18, 1.60]	0.61 [0.32, 2.09]	0.39 [0.21, 2.97]	0.73 [0.38, 1.18]	0.36
CER C16:0 (ng/mgCr)	-	2.16 [1.20, 7.96]	3.25 [0.93, 11.57]	3.58 [1.08, 7.12]	4.35 [2.85, 10.32]	0.088
CER C18:0 (ng/mgCr)	-	0.41 [0.23, 1.21]	0.72 [0.19, 1.86]	0.61 [0.29, 1.58]	0.80 [0.59, 1.87]	0.189

CER C18:1 (ng/mgCr)	-	0.10 [0.04, 0.37]	0.13 [0.04, 0.39]	0.12 [0.06, 0.61]	0.19 [0.10, 0.47]	0.194
CER C20:0 (ng/mgCr)	-	0.58 [0.27, 1.40]	1.21 [0.25, 3.42]	1.00 [0.49, 1.89]	0.85 [0.55, 1.96]	0.278
CER C22:0 (ng/mgCr)	-	1.22 [0.49, 3.00]	1.53 [0.39, 7.22]	1.70 [0.59, 4.72]	1.99 [1.28, 3.40]	0.171
CER C24:0 (ng/mgCr)	-	2.51 [1.35, 5.44]	2.28 [0.81, 9.52]	3.64 [1.16, 8.32]	5.20 [3.81, 7.72] †	0.032

Data was presented as median [interquartile range] for the -variables.

Abbreviations: CER, ceramide; dhS1P, dihydrosphingosine 1-phosphate; dhSph, dihydrosphingosine; S1P, sphingosine 1-phosphate.

*: $P < 0.05$ vs. control, †: $P < 0.05$ vs. chronic glomerulonephritis, ‡: $P < 0.05$ vs. diabetes mellitus, §: $P < 0.05$ vs. arterial hypertension.
